# Correction to: Unique arm-flapping behavior of the pharaoh cuttlefish, *Sepia pharaonis*: putative mimicry of a hermit crab

**DOI:** 10.1007/s10164-017-0538-4

**Published:** 2017-12-01

**Authors:** Kohei Okamoto, Haruhiko Yasumuro, Akira Mori, Yuzuru Ikeda

**Affiliations:** 1 Department of Zoology, Graduate School of Science, Kyoto University, Kitashirakawa Oiwake-cho, Sakyo, Kyoto, 606-8502 Japan; 2 Present Address: Department of Chemistry, Biology and Marine Science, Faculty of Science, University of the Ryukyus, 1 Senbaru, Nishihara, Okinawa 903-0213 Japan; 3 Division of Marine and Environmental Sciences, Graduate School of Engineering and Science, University of the Ryukyus, 1 Senbaru, Nishihara, Okinawa 903-0213 Japan

## Correction to: J Ethol (2017) 35:307–311 10.1007/s10164-017-0519-7

The article, “Unique arm-flapping behavior of the pharaoh cuttlefish, Sepia pharaonis: putative mimicry of a hermit crab”, written by Kohei Okamoto, Haruhiko Yasumuro, Akira Mori and Yuzuru Ikeda, was originally published Online First without open access. After publication in volume 35, issue 3, pages 307–311 the author decided to opt for Open Choice and to make the article an open access publication. Therefore, the copyright of the article has been changed to © The Author(s) 2018 and the article is forthwith distributed under the terms of the Creative Commons Attribution 4.0 International License (http://creativecommons.org/licenses/by/4.0/), which permits use, duplication, adaptation, distribution and reproduction in any medium or format, as long as you give appropriate credit to the original author(s) and the source, provide a link to the Creative Commons license, and indicate if changes were made.

